# Perfecting the pour: A novel co-axial technique with sequential injections for optimising cement delivery during sacroplasty

**DOI:** 10.1177/15910199241282709

**Published:** 2024-09-13

**Authors:** Mehran Nasralla, Afra Alfalahi, Eef J Hendriks, Kieran Murphy, Roger Smith

**Affiliations:** 1 Division of Neuroradiology, Joint Department of Medical imaging, 26625 Toronto Western Hospital, University of Toronto, Toronto, Ontario, Canada

**Keywords:** Percutaneous sacroplasty, co-axial sacroplasty, sequential injection, sacral insufficiency fractures, cement leakage

## Abstract

**Background:**

Percutaneous sacroplasty is an effective treatment for painful sacral fractures and tumours, however there is no accepted optimal technique for performing this procedure. This study investigated a novel approach to sacroplasty combining co-axial sacral access, sequential cement injections and hypothermic cement manipulation to improve cement delivery.

**Methods:**

This retrospective study analysed 11 patients who underwent co-axial sacroplasty between April 2023 and March 2024 for treatment of painful insufficiency fractures (*n* = 5) or malignant sacral tumours (*n* = 6). All cases were performed using biplane fluoroscopy with conebeam CT navigation for planning and monitoring percutaneous access. Procedural details, technical outcomes, and clinical outcomes including Numerical Rating Scale (NRS) pain and analgesic utilisation on a six-point scale were analysed pre-procedure and at follow-up.

**Results:**

Technical success of was achieved in all cases using this technique. The mean injected cement volume was 20.5 ± 6.4 ml. Median pre-procedural NRS pain scores of 8 (IQR 7.25–8) significantly decreased to 0 (IQR, 0–0.25) at follow-up (*p* <.01). The median preprocedural analgesic utilisation score reduced from 3 (IQR, 2–3) to 0 (IQR, 0–2.5) at follow-up (*p* <.01). Cement leakage occurred during two cases without associated adverse clinical sequelae. There were no major adverse events.

**Conclusion:**

Co-axial sequential injection sacroplasty is a safe and effective technique which allows facilitates controlled delivery of cement. Improved control of cement delivery, including around high-risk structures for cement leakage, offers a potential safety advantage over conventional sacroplasty techniques. Further research comparing technical and clinical outcomes to conventional techniques is warranted.

## Background

Percutaneous sacroplasty is an effective treatment for ameliorating debilitating pain associated with insufficiency fractures or malignant lesions. This procedure initially evolved from vertebroplasty procedures whereby polymethyl methacrylate (PMMA) bone cement is injected percutaneously into sacrum. In contrast to vertebroplasty procedures however, there is no accepted optimal technique for performing sacroplasty. The sacrum presents specific technical challenges towards both safe access and cement delivery. The neural foramina and spinal canal are vulnerable to transgression during percutaneous access and their integrity is often compromised by fractures and tumours, increasing the risk of cement leakage (or extravasation) which can cause neurological deficits. While adverse events from cement leaks are uncommon, they may require additional treatment including nerve root injections^1^ or rarely, surgical extraction.^2–4^ Furthermore, achieving satisfactory cement distribution can be challenging due to the relatively large size of the sacral intramedullary space, longitudinally extensive configuration of sacral ala insufficiency fractures typically encountered, and large volume tumours.^4^ If early cement leakage is encountered, this can preclude further injection and achieving adequate cement distribution.

Various techniques have been described for sacral access. Short-axis approaches target the sacral ala, avoiding the sacral foramina at the expense of treating centrally located lesions.^5–7^ Long-axis and transiliac techniques allow for contiguous cement distribution in longitudinal and axial planes respectively.^8,9^ These approaches allow treatment of fractures and lesions in the sacral body or close to the neural foramina; however, the potential for leakage is greater in these areas. Furthermore, there is a risk of unintended displacement of unpolymerised injected cement upon subsequent injections leading to cement leakage, particularly with transiliac and long axis approaches as the sacroplasty needle is withdrawn along the lengthier needle tract (Figure 1). Unintended propagation of cement has been reported in vertebroplasty literature,^10,11^ with the effect compounded in the sacrum due to the length of needle tracts. Furthermore, there has been little discussion in the literature regarding the method and timing of cement injection.

**Figure 1.**
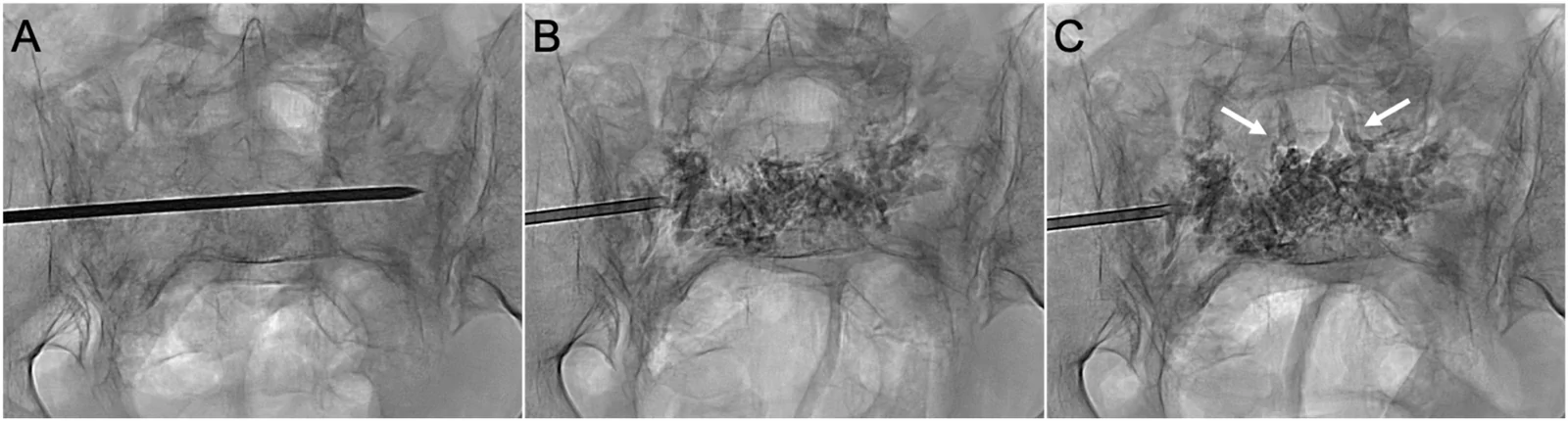
Epidural venous cement extravasation during conventional sacroplasty for bilateral sacral ala insufficiency fractures. (A) PA radiograph shows a transiliac approach at S1. The needle was inserted via the left sacral ala, traversing the sacral body to access the contralateral ala. (B) PA radiograph shows the cement distribution across the sacrum as the needle was withdrawn. (C) Epidural venous plexus extravasation *(arrows)* occurred following additional cement injections in the ipsilateral ala. This resulted from unintended cement propagation along the needle tract, rather than dispersing in the sacral ala around the needle tip.

We developed a novel approach to percutaneous sacroplasty to improve the control of cement delivery which combines co-axial sacral access with sequential cement injections and hypothermic manipulation of bone cement. A co-axial system utilising an outer guide cannula and repositionable inner injection cannula allows multiple cement injections over a planned trajectory. Sequential cement injections have been shown to reduce cement displacement and leakage during vertebroplasty by exploiting differential rates of cement polymerisation between injected and uninjected cement.^11,12^ Similarly, hypothermic manipulation of cement allows the operator to further exploit this principle by slowing the polymerisation of uninjected cement.^13,14^ The aim of this study was to investigate the clinical and technical outcomes of this co-axial sacroplasty technique. The study objective was to achieve satisfactory cement distribution over the sacral target regions without incurring adverse events, including unintended distal cement displacement, whilst achieving clinically meaningful pain relief. We hypothesise that this technique will result in consistently larger volumes of injected cement without increasing the rate of adverse events.

## Methods

### Patient selection

This retrospective study was approved by our institutional review board. Eleven patients who underwent co-axial sacroplasty from April 2023 to March 2024 were identified from our database of percutaneous spine augmentation procedures. Patients treated for sacral fractures or malignant sacral lesions with pain unmanaged by conventional therapy (immobilisation, opioids, and radiotherapy) were included. Demographic and clinical data were collected from retrospective chart review. Primary cancer diagnoses were recorded where relevant. Radiological data, including fracture aetiology, fracture or lesion location, and cortical breach resulting from fracture or tumour involvement (neural foramina, spinal canal, sacroiliac joint, or anterior alar cortex) were recorded. The distribution of fractures and malignant lesions were additional described as per the Denis classification:^15^ Zone I, sacral ala; Zone II, foraminal region; Zone III, involving the sacral body. The exclusion criteria included conventional (non-co-axial) sacroplasty cases or prior sacroplasty. Clinical assessment and examination were performed pre-procedure (outpatient clinic, or inpatient unit) and at approximately one-month post-procedure.

### Co-axial technique

Written informed consent was obtained from all patients prior to the procedure. All procedures were performed by two radiologists experienced in spine augmentation procedures. Patients were positioned in a prone position with bolsters on the angiography suite table. All procedures were performed under conscious intravenous sedation with anaesthetic monitoring. Prophylactic intravenous antibiotics were administered at the start of each case, and strict aseptic technique was followed. A biplane fluoroscopy machine with conebeam CT capabilities (Allura Xper FD20/20, Philips Healthcare) was used to acquire a CT for planning needle trajectories and 3D needle navigation (XperGuide). The planned trajectories were based on the location of sacral fractures or tumours. The lateral (transiliac) approach could be used to target fractures or lesions in either the sacral body or ala, while short and long-axis approaches could target the sacral ala (Figure 2). Multiple needle trajectories were planned when required for treatment of the target(s). Local anaesthetic was infiltrated into the skin and deepened to the periosteum at the entry point. Following skin incision, the co-axial guide and injection cannula were inserted into the sacrum along the planned trajectory (10G diamond-tipped guide needle, 11G end-opening or side-opening injection cannula, Confidence, DePuy Synthes; or 11G diamond-tipped needle, 14G biopsy needle, Murphy M2S, IZI Medical). The guide cannula distal opening was positioned beyond the sacral alar cortex, and for transiliac approaches, medial to the sacroiliac joint space. The guide cannula distal opening was positioned partway along the planned path, clear of the sacral cortex and sacroiliac joint space. The injection cannula was then inserted co-axially through the guide cannula and advanced to the distal target. An alternative technique involved advancing the guide cannula to the distal target first. The stylet is then replaced with the injection cannula before the guide cannula is retracted into the desired proximal position. A bone drill was used in cases where the injection cannula did not have a cutting tip (side-opening cannula). Short axis needles, when utilised, were inserted using a conventional technique without use of a co-axial guide cannula. Real time monitoring of progress was performed using fluoroscopy with 3D navigation (XperGuide).

**Figure 2.**
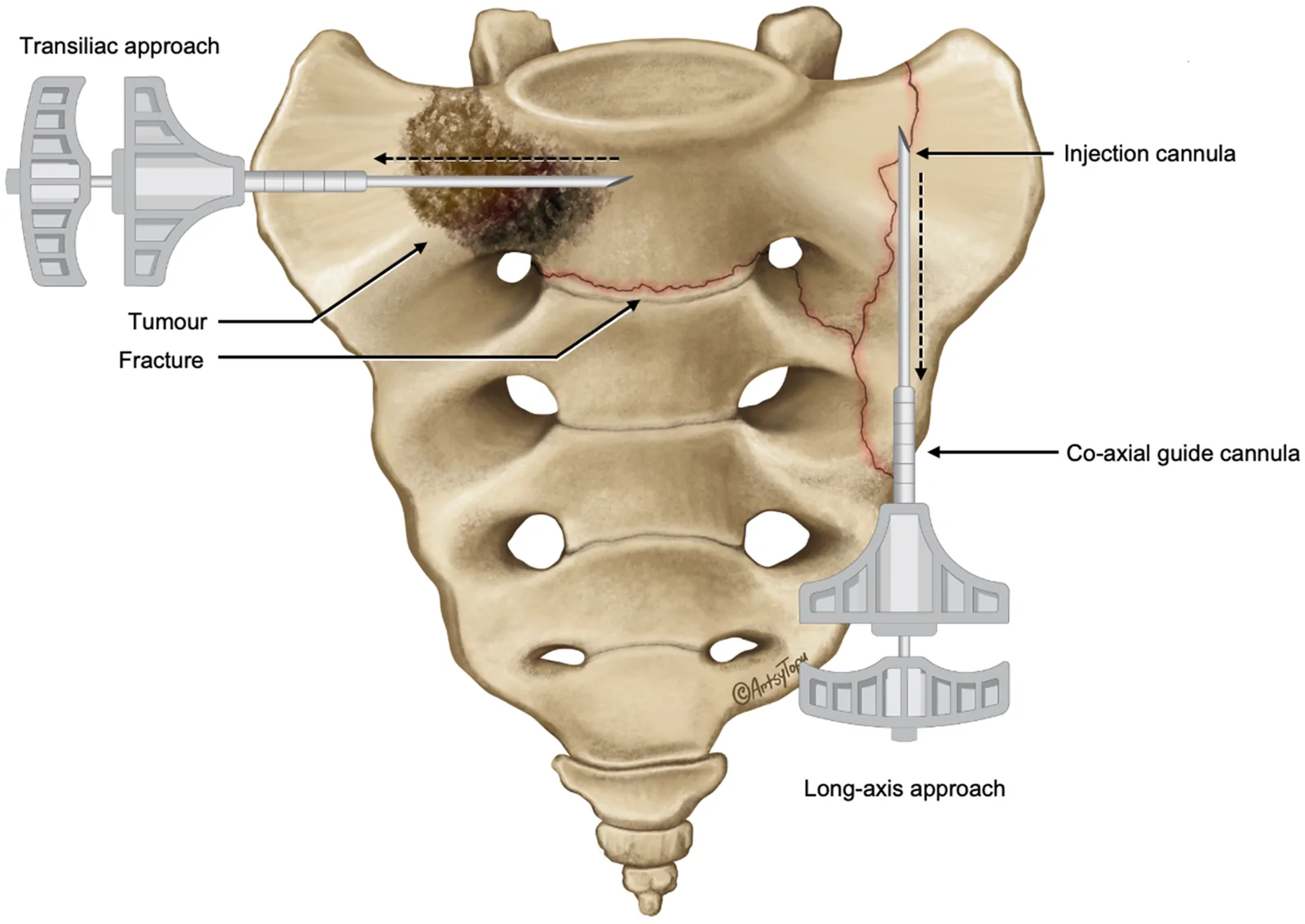
Illustration of the positions of the outer co-axial guide and inner injection cannulas for transiliac and long-axis approaches. The outer guide cannula allows easy repositioning of the injection cannula across the target regions (dashed line arrows) to facilitate sequential cement injections while the guide cannula maintains sacral access between injections.

### Cement delivery technique

PMMA bone cement (Kyphon HV-R, Medtronic) was prepared and stored on ice to prolong the working time and injected into the sacrum after reaching an appropriate viscosity using precooled DSMO syringes or proprietary delivery systems (Kyphon CDS, Medtronic). Cement injections were performed sequentially through the injection cannula, with initial aliquots injected into the distal target until satisfactory cement distribution was achieved. Subsequent injections were performed at 2-to-3-min intervals, allowing previously injected cement to polymerise before additional aliquots are injected. Between injections, the injection cannula was repositioned by withdrawal into an untreated region. This process was repeated until a satisfactory and contiguous cement distribution was achieved in the desired regions (Figure 3). For cases requiring bilateral augmentation, alternating sequential injections between the left and right sides during each interval period helped minimise procedure time and facilitated polymerisation between ipsilateral injections (Figure 4). Continuous fluoroscopic monitoring was performed to assess cement distribution and detect cement leakage or unintended distal cement displacement. Cement injection was halted if either were detected, and subsequent injections carefully performed after an additional interval to allow additional curing of injected cement and injection cannula repositioning.

**Figure 3.**
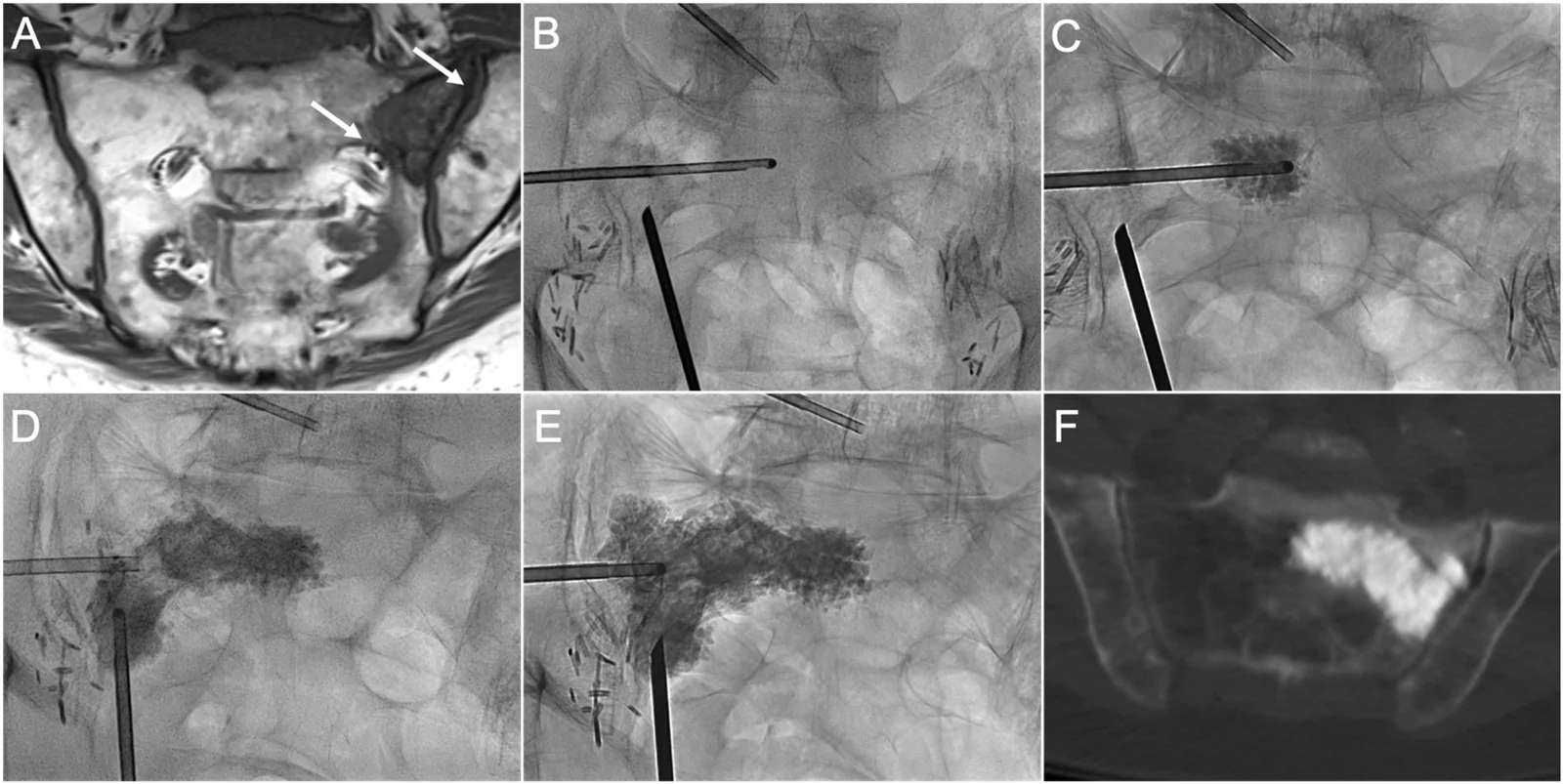
Co-axial sacroplasty of a left sacral ala metastasis. (A) Coronal T1 MRI image shows left ala metastatic lesion with erosions of the sacroiliac joint and S1 foraminal cortical margins (*white arrows*), which increase the risk of cement leakage. (B) PA fluoroscopic image demonstrates transiliac and long-axis guide cannula positioning. A side-opening injection cannula was inserted co-axially through the transiliac guide cannula across the lesion. (C) PA radiograph demonstrates the initial cement deposition. (D) Cement was injected through the long-axis guide cannula after initial sequential transiliac injections were completed. (E) Final distribution of cement after completion of long-axis and an additional transiliac injection in the ala superiorly. (F) Axial CT image shows contiguous cement distribution without evidence of foraminal or sacroiliac joint extravasation.

**Figure 4.**
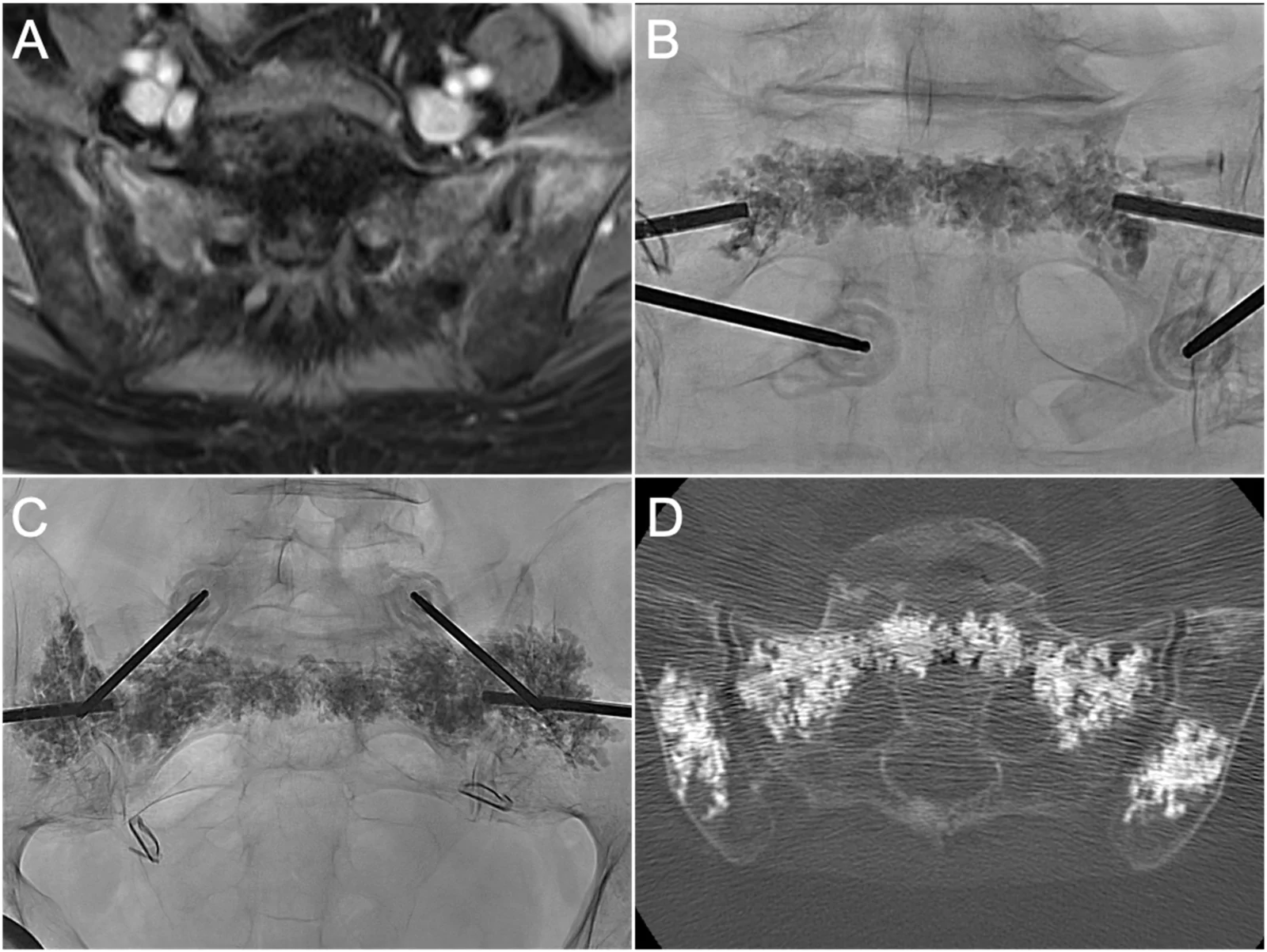
Co-axial sacroplasty and iliac cementoplasty for bilateral sacral and iliac insufficiency fractures, in a patient previously treated with pelvic radiotherapy for cervical cancer. (A) Axial T1FS with gadolinium demonstrates contrast enhancement in the sacral ala and iliac bones consistent with insufficiency fractures. (B) PA radiograph demonstrates cement distribution in the sacral body (Zone I) and periforaminal regions (Zone II) after sequential injections. (C) PA radiograph shows contiguous cement distribution from ala-to-ala at S1 following sequential cement injections, and bilateral iliac cementoplasties. (D) Axial CT image shows cement overall distribution and absence of cement leakage.

### Outcomes

Technical outcomes recorded included sacroplasty approach (transiliac, long-axis, short-axis), treatment laterality, sacral levels treated, total cement volume injected, and number of needles used. Technical success was defined as satisfactory cement distribution around the plane of a fracture or throughout the area of tumour involvement. Instances of cement leakage were recorded according to anatomical location. Clinical outcomes included patient reported pain intensity scores and analgesic utilisation. Pain intensity was assessed on the 11-point numerical rating scale (NRS), where from 0 represents no pain and to 10 represents the worst pain imaginable.^16^ Analgesic utilisation was graded on a 6-point Analgesic Scale described by Gupta et al.: 0 (*no pain medication*), 1 (*aspirin, acetaminophen, non-steroidal anti-inflammatory drugs*), 2 (*prescription non-opioids*), 3 (*oral opioids, as required*), 4 (*oral opioids, scheduled*), 5 (*parenteral opioids*).^17,18^ Pain scores and analgesic grades were assessed pre-procedurally at approximately 1-month follow-up. Adverse events were classified based on the Society of Interventional Radiology guidelines.^19^

### Statistical analysis

Results were reported as mean ± standard deviation for parametric data or median (range or interquartile range) for non-parametric data. The Wilcoxon signed-rank test was used for comparison of pre- and post-sacroplasty pain and analgesic scores. Statistical significance was set at *p* < .05. Statistical analysis was performed using SPSS, version 29.0 for MacOS (IBM SPSS Statistics, Armonk, NY: IBM Corp).

## Results

The study cohort consisted of eleven patients who underwent co-axial sacroplasty between April 2023 and March 2024. Demographic and imaging data are shown in Table 1. Indications for treatment included sacral insufficiency fractures (5/11) and tumours (6/11). Amongst the patients with insufficiency fractures, three were osteoporotic, with two associated with prior radiotherapy (rectal cancer). In the malignant tumour group, primary cancer diagnoses included myeloma (3/6), and metastatic breast (1/6), thyroid (1/6), prostate (1/6) cancers. Most of the tumour cases (5/6) were treated with prior radiation. All malignant lesions were osteolytic. Among the entire cohort, cortical breach involving at least one anatomic site was present in 90.9% (10/11) of patients, most frequently involving the neural foramina in nine patients (81.8%) and anterior alar cortical margins in four patients (36.4%). Five patients had two anatomic sites of cortical breach, while one patient had three separate sites.

**Table 1.** Clinical and radiological characteristics of co-axial sacroplasty patients.

Characteristics	Data
Total patients	11
Age, median (range)	70 (55–77)
Female/male ratio	6/5
Inpatient/outpatient	2/9
**Sacral fractures**	
Osteoporosis	3 (27.3%)
Radiation-associated	2 (18.2%)
Unilateral/bilateral fractures	1/4
**Sacral tumours**	
Metastasis	3 (27.3%)
Myeloma	3 (27.3%)
**Cortical breach**	
Neural foramina	9 (81.8%)
Sacral canal	1 (9.1%)
Anterior alar	4 (36.4%)
Sacroiliac joint	2 (18.2%)
S1 endplate	1 (9.1%)

The technical approach and clinical outcomes are reported in Table 2. A transiliac-based approach was used in 10 (90.9%) patients: exclusive transiliac approach in six patients (54.5%), combined co-axial long-axis approach in two patients (18.2%), or combined conventional short axis approaches (18.2%) in two patients. A bilateral co-axial long-axis technique was utilised in one patient (9.1%). Bilateral treatments were performed in seven patients (66.6%), reflecting lesion distribution. The most frequently treated sacral levels were S1 (5/11), followed by S1–S2 (4/11), S2 (1/11), and S1–S3 (1/11). Technical success, defined as adequate cement filling of the target lesion or around the fracture plane, was achieved in all cases. The average cement volume injected into the sacrum was 20.5 ml ± 6.4 (range, 7–30 ml). The median number of needles used for sacral access was two (range, 1–4). Concomitant vertebroplasty or iliac cementoplasty was performed in 63.6% (7/11) of patients; vertebroplasty (5/11), iliac cementoplasty (1/11), and vertebroplasty with iliac cementoplasty (1/11). The median number of vertebral levels treated was 1 (range, 1–2). The median procedural duration was 78 min (range, 33–99 min). Cement leakage occurred during two procedures (18.2%). One case included presacral venous leakage and foraminal leakage, latter related to fracture involvement of the foramina. Foraminal cement leakage did not propagate on later sequential injections after initially injected aliquots of cement had cured (Figure 5). Epidural cement leakage occurred in another case. No associated neuropathy was reported post-procedure or at follow-up in either case.

**Figure 5.**
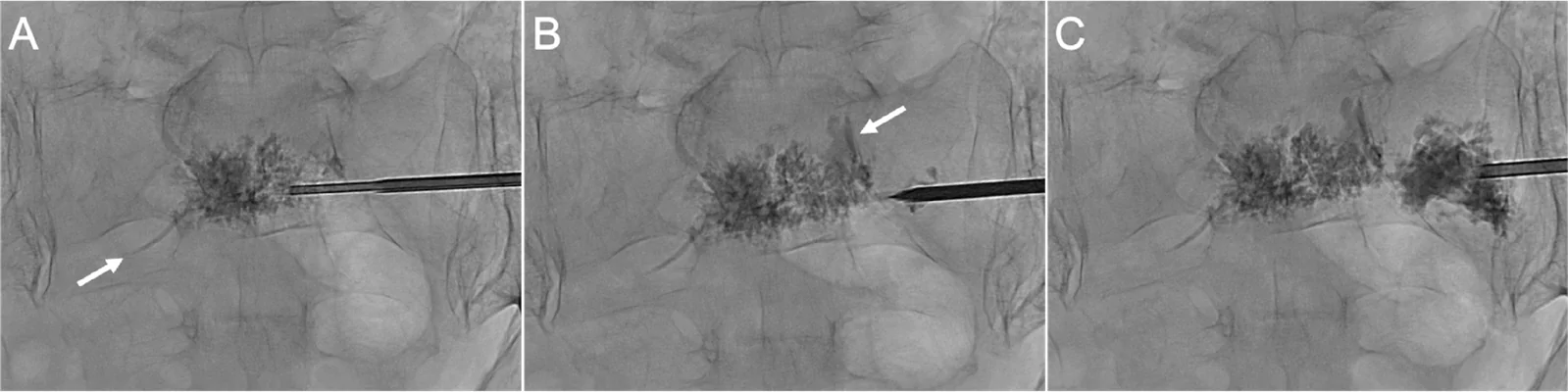
First application of the co-axial sequential injection technique involving a patient with insufficiency fractures involving the sacral body and right foramen. (A) PA radiograph after initial injection demonstrates cement in the sacral body and small presacral venous extravasation (*arrow*). (B) Subsequent injection around the foramen showed a small foraminal leakage (*arrow*). (C) Subsequent injection, following a period for cement curing, showed that the foraminal leak remained unchanged (*arrow*) without further propagation along the needle path. The foraminal leak was asymptomatic.

**Table 2.** Characteristics, procedural details, and outcomes of patients who underwent co-axial sacroplasty.

Patient				Technique				Pre-procedure		Post-procedure	
Case	Age (years)	Sex	Diagnosis Location of lesion(s)	Approach (laterality) level	Guide/injection cannula cement injector type	Cement volume injected (ml)	Cement leakage	NRS	Analgesic utilisation	NRS	Analgesic utilisation
1	74	F	Fracture (osteoporosis). Zone II and III fractures	Transiliac (unilateral) S2	11G-guide/14G-injection cannula. DSMO syringes.	7	*Y	8	1	0	0
2	70	F	Multiple myeloma Zone III lytic lesion	Transiliac (unilateral) S1	11G-guide/14G-injection cannula. DSMO syringes.	14	N	2	3	0	0
3	55	F	Thyroid cancer Left Zone I/II lesion	Transiliac + long-axis (unilateral) S1 + S2	10G-guide/11G-injection cannula (side-opening). Kyphon CDS.	22	N	7	4	0	4
4	62	M	Multiple myeloma Zone III lesion	Transiliac (bilateral) S1	10G-guide/11G-injection cannula (side-opening). Kyphon CDS.	26	N	8	1	0	1
5	58	F	Fracture (radiation-associated) Bilateral Zone I fractures	Transiliac (bilateral) S1	10G-guide/11G-injection cannula (side-opening). Kyphon CDS.	24	N	10	3	0	0
6	76	F	Breast Ca Zone III lytic lesion	Transiliac (bilateral) S1	10G-guide/11G-injection cannula (side-opening/end-opening). Kyphon CDS.	24	**Y	8	4	0	0
7	74	M	Prostate Ca Left Zone I lytic lesion with pathological fracture	Transiliac + long-axis (unilateral) S1 + S2	10G-guide/11G-injection cannula (side-opening). Kyphon CDS.	18	N	8	1	1	0
8	65	F	Fracture (osteoporosis) Bilateral Zone I fractures	Long-axis (bilateral) S1–S3	10G-guide/11G-injection cannula (side-opening). Kyphon CDS.	18	N	10	4	^+^N/A	^+^N/A
9	60	M	Multiple myeloma Bilateral Zone I lytic lesions	Transiliac (bilateral) S1	10G-guide/11G-injection cannula (side-opening). Kyphon CDS.	18	N	8	3	0	0
10	77	M	Fracture (osteoporosis) Bilateral Zone I fractures + Zone III (transverse) fractures	Transiliac + short-axis (bilateral) S1 + S2	10G-guide/11G-injection cannula (side-opening/end-opening). Kyphon CDS.	25	N	9	3	1	3
11	75	M	Fracture (radiation-associated) Bilateral Zone I and II fractures + Zone III (transverse) fractures	Transiliac + short-axis (bilateral) S1 + S2	10G-guide/11G-injection cannula (end-opening). Kyphon CDS.	30	N	6	5	4	5

* Presacral venous extravasation. Right S2 foraminal cement leak associated with cortical breach (Figure 5).

** Epidural leak associated with cortical breach.

+ Patient lost to follow up.

The median pre-procedural NRS was 8 (IQR, 7.25–8) which significantly decreased by 8 points to 0 (IQR, 0–2.5) at follow-up (*p* < .01) (Table 3). At follow-up, ten patients (90.9%) had NRS scores < 4, indicating no pain or mild pain. Similarly, the median analgesic utilisation score significantly reduced by 3 points from 3 (IQR, 2–4) to 0 (IQR, 0–2.5) (*p* < .01). Six patients did not require any analgesics at follow-up, while analgesic utilisation was unchanged in four patients (median, 3.5; IQR, 2.5–4.25). The median interval from pre-procedural evaluation to sacroplasty was 8 days (IQR, 7–12), and the median interval from sacroplasty to follow-up was 5.4 weeks (IQR, 4.2–6.8). Most procedures (81.8%, 9/11) were performed as day cases. Two inpatients underwent sacroplasty (Cases 8 and 11; Table 2). The first of these was discharged two days post-procedure after early improvements in pain but was subsequently lost to follow-up. The second patient had extended hospitalisation for ongoing oncological treatments and was discharged one-month post-procedure. Modest improvements in clinical outcomes at follow-up were potentially confounded by locally advanced rectal tumour-associated pain. No adverse events, including neuropathy, infection, haemorrhagic events, or other symptomatic complications, were reported.

**Table 3.** Outcome data for patients who underwent co-axial sacroplasty.

Outcome	Pre-procedure	Post-procedure	Change	*p*-value
Numerical Rating Scale pain score (median, IQR)	8 (7.25–8)	0 (0–2.5)	−8	<.01
Analgesic utilisation score (median, IQR)	3 (2–4)	0 (0–2.5)	−3	<.01

## Discussion

Percutaneous sacroplasty has emerged as an effective treatment for painful sacral fractures and tumours. Initial studies evaluated its role in the treatment of osteoporotic insufficiency fractures,^5,20^ although it has subsequently be shown to be a viable treatment option for pain palliation in patients with cancer-related fractures^17,21^ and malignant tumours and metastases^2,4,22^^–24^. However, there is no universally accepted optimal technique for performing sacroplasty, in contrast to the well-established methods for vertebral augmentation. This study aimed to investigate the feasibility, safety, and efficacy of a novel sacroplasty technique that combines co-axial sacral access, sequential cement injections, and hypothermic manipulation of bone cement. Our results demonstrate that this technique can help consistently delivery sufficient cement volumes over the target regions in the sacrum, including those with risk factors for cement leakage, without incurring a significant increase in cement leakage or adverse events. The reduction in pain scores and analgesic utilisation at short-term follow-up is consistent with the outcomes reported in other studies.

Sacroplasty techniques utilising both co-axial access and sequential injections have not been previously described in the literature. While co-axial access has been described in sacroplasty case reports and studies for specific purposes such as biopsy,^25^ radiofrequency ablation,^4^ or cavity creation,^9^ our study is the first to employ it as a means to maintain sacral access while allowing for controlled cement injection with a repositionable cannula. This approach enables the operator to optimise cement delivery and minimise the risk of leakage. The consistency of delivered cement volumes and overall procedural duration suggests a relatively short learning curve to those familiar with spine augmentation procedures.

The overall rate of cement leakage of 18.2% in our study is within the range of similar published studies (6.8–58.1%).^21,22,26^ In addition to technical factors, the different rates reported may in part be due to differences in the underlying pathology and associated cortical defects which promote cement leakage. This is supported in a recent study specifically examining leakage rates between osteoporotic fractures and neoplastic lesions, which reported a leakage rate of 13% and 45% respectively.^27^ Sequential cement injection has suggested as a method to mitigate this mode of leakage, having been shown to reduce the rate of cement leakage in the vertebroplasty literature.^11,12^ Hoppe et al. demonstrated a significantly reduced rate of leakage with an injection interval of one minute in vertebroplasty models compared to single-phase injections.^12^ The proposed mechanism for reducing cement leakage is the sealing of potential leakage pathways through cement curing before additional aliquots are injected.^11,28^ Our results support this as we were able to avoid cement leakage in most which had cortical defects while delivering larger cement volumes compared to other studies. While the two cases of cement leakage were associated with cortical defects, subsequent injections did not increase the leakage, suggesting that initial cement injections sealed the route of leakage. These cement leaks were not associated with adverse clinical sequelae. Additionally, we found that side-opening cannulae, which have been utilised in vertebroplasty studies,^29,30^ were helpful in directing cement away from high-risk areas for leakage in some cases (Figure 6). Overall, these findings indicate that it is feasible to inject cement in cases which risk factors for cement leakage are present, including cortical defects related to the neural foramina. Nonetheless, we emphasise the importance and need to identify risk factors for leakage during patient workup, and to exercise caution when injecting cement in cases which have a greater potential for leakage.

**Figure 6.**
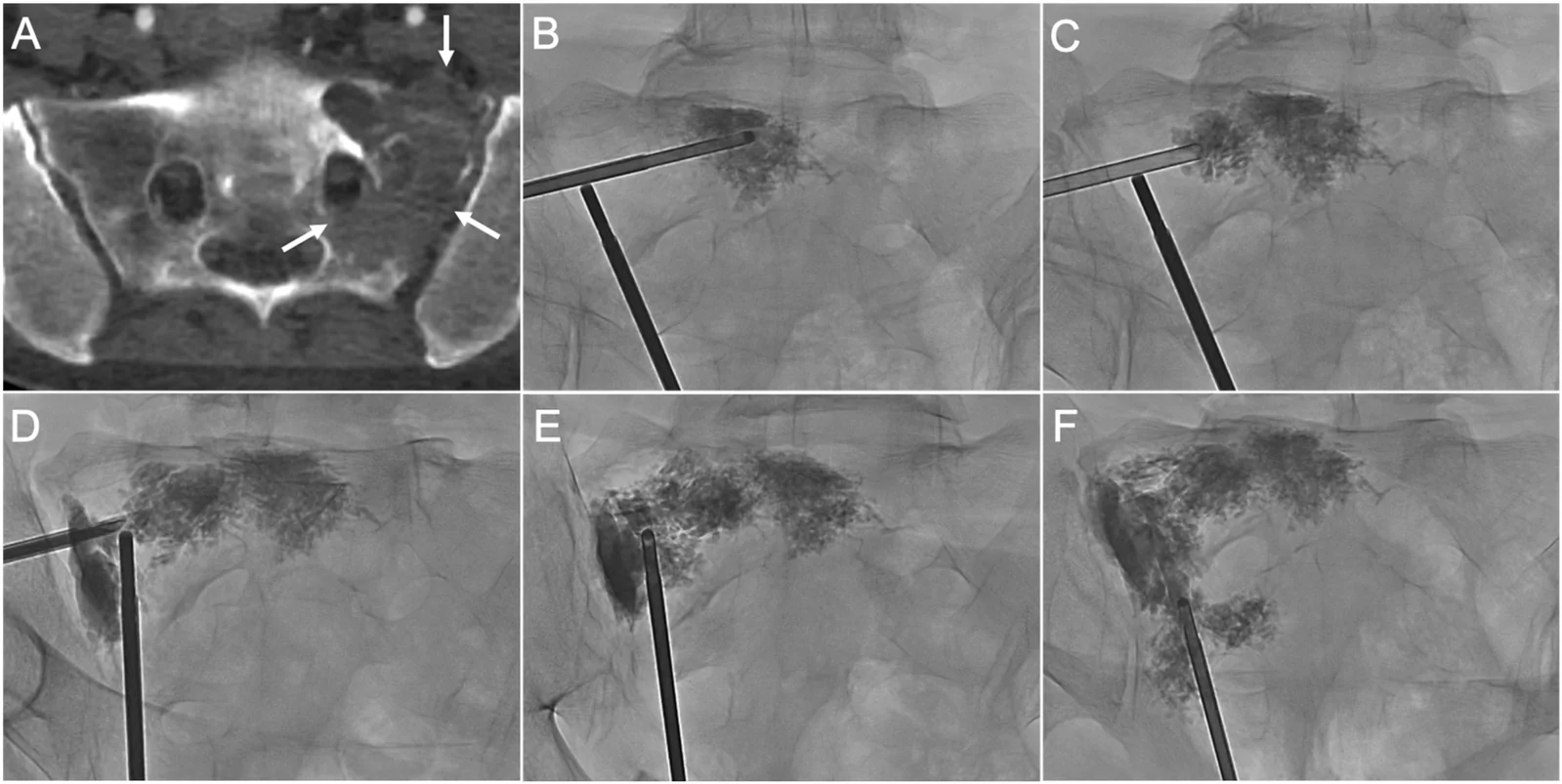
Application of side-opening injection cannulae in the treatment of complex sacral lesions. (A) Axial CT image demonstrates extensive osteolytic disruption of the left S1 foraminal, sacroiliac joint, and anterior sacral margins (*arrows*). (B–D) PA radiographs demonstrate sequential cement injections via the transiliac approach from the sacral body to the ipsilateral ala. (E–F) AP radiographs after long-axis sequential injections filled the remaining areas in the sacral ala to achieve a contiguous distribution. The side-opening was directed away from the foramen during cement injections. No foraminal, sacroiliac joint or extraosseous leakage was demonstrated.

The 2-to-3-min interval between injections in our study was selected based on our clinical experience with our cement of choice and its working and setting times.^31^ This appeared sufficient for preventing unintended cement displacement, although the optimal timing may vary significantly depending on type of PMMA utilised and use of hypothermic manipulation. Bone cements with a longer setting time may remain liquid within the bone for longer and thus may not sufficiently cure to prevent further displacement upon sequential injections.^10^ Hypothermic manipulation, which has also been described in vertebroplasty literature^14,32^ aids sequential injections by extending the working time of uninjected cement and allowing additional time for injected cement to sufficiently cure between injections to prevent unintended displacement. Bone cements with shorter setting times when matched with hypothermic manipulation are ideal for this procedure, facilitating faster curing following injection with an extended working time required for multiple sequential injections.

Our technique proved particularly suited to transiliac and long-axis approaches which involve long intraosseous needle tracts. These tracts offer a path of least resistance following needle repositioning which may lead to unintended cement propagation if the injected cement is insufficiently cured. The co-axial sequential injection system effectively mitigated this risk in our study, as we did not observe further cement displacement into earlier injected regions or leakage along the needle tracts. Furthermore, the co-axial system preserved sacral access between injections in all our cases as the external guide cannula remained cement-free.

The mechanism by which sacroplasty reduces pain remains a subject of debate. Both thermo-ablative effects and cytotoxic properties of PMMA on intraosseous nerves and tumour cells,^33^ as well as reducing fracture micromotion have been cited.^34^ Biomechanical studies have yielded mixed results regarding the impact of sacroplasty on overall sacral stiffness and stress^34^^–37^. Finite element analyses have suggested that cement augmentation reduces mechanical stress and micromotion in sacral fractures,^34^ the effects are highly localised to the areas of cement deposition.^35^ Cadaveric studies did not demonstrate significant increases in overall pelvic strength or stiffness,^36,37^ with no effect of variable cement volumes.^37^ However, in these studies, cement distribution was generally confined to the sacral ala in a non-contiguous distribution. Anderson et al. suggests that a greater fill fraction may thus be required to increase overall stiffness in the sacrum,^35^ similar to prior vertebroplasty models which have shown that cement significantly increases vertebral stiffness.^38^ PMMA has been postulated to provide better resistance against shearing and compressive forces if delivered in a contiguous distribution.^8^ Cement augmentation over a larger distribution in areas of bone loss^39,40^ might improve overall stiffness and potentially avoid transmitting forces into non-augmented adjacent segments of weakened bone,^35,36^ although this remains to be proven experimentally. Further biomechanical studies investigating the relationship between cement volume and distribution in sacroplasty would be of interest for future research. While these biomechanical implications require further investigation, delivering cement over a target distribution without premature termination of cement injection due to leakage nonetheless remains desirable from an operator standpoint.

The main limitations of our study include its retrospective design, small sample size, lack of a control group, and short follow up period. Two patients underwent a combined approach with co-axial and conventional techniques which could confound our outcomes, although the conventional short-axis approaches encompassed a relatively smaller fraction of the overall volume of injected cement. We also acknowledge that our study design does not allow for a direct comparison of technical outcomes with conventional sacroplasty techniques. Further research with a larger sample size, additional follow-up intervals, and patient-reported outcome measures would provide more robust evidence to support the efficacy of this technique in comparison to conventional techniques.

## Conclusion

This study demonstrates the feasibility, safety, and efficacy of a novel sacroplasty technique that combines co-axial access, sequential injections, and hypothermic manipulation of bone cement. Improved control of cement delivery, including around high-risk structures for cement leakage, offers a potential safety advantage over conventional sacroplasty techniques. Further research comparing clinical and technical outcomes to conventional techniques is warranted.

## Data Availability

All data generated or analyzed during this study are included in this published article.
